# Oral Manifestations in Monkeypox: A Scoping Review on Implications for Oral Health

**DOI:** 10.3390/dj11050132

**Published:** 2023-05-12

**Authors:** Asmaa Wajeeh Issa, Nada Fayyad Alkhofash, Divya Gopinath, Sudhir Rama Varma

**Affiliations:** 1 Department of Clinical Sciences, Ajman University, Ajman P.O. Box 346, United Arab Emirates; asmawajihissa12@gmail.com (A.W.I.); nada_fayyad.k@hotmail.com (N.F.A.); 2 Department of Basic Sciences, Ajman University, Ajman P.O. Box 346, United Arab Emirates; d.gopinath@ajman.ac.ae; 3 Center for Medical and Bio-Allied Health Sciences Research, Ajman University, Ajman P.O. Box 346, United Arab Emirates

**Keywords:** monkeypox, Mpox, poxvirus, zoonotic orthopox DNA virus, sore throat, ulcers, vesicles

## Abstract

Background: The monkeypox outbreak in 2022 caused concern in the public. Infected patients usually present prodromal symptoms, such as lesions on their skin and mucous membranes, including the oral cavity. The current study aims to review the most common oral/perioral manifestations reported to date. Methods: A literature search was conducted in the PubMed, Research Gate, and Wiley Online Library databases, as well as in the Google search engine, using keywords related to the condition. Of the 56 publications identified, 30 were selected, including 27 case reports, two case series types, and one cross-sectional study published from 2003 to 2023 in endemic and non-endemic countries. Of the 54 patients in these studies, data on the oral symptoms and sites of monkeypox were interpreted from 47 patients. Results: Oral/perioral signs as one of the initial manifestations were reported in 23 out of 47 patients (48.93%). Out of the 47 patients with oral/perioral involvement, the most common signs/symptoms were sore throat, followed by ulcers, vesicles, dysphagia and odynophagia, and erythema. Conclusion: The most common oral symptom of monkeypox is sore throat, followed by ulcers. The symptoms usually occur in the pharynx/oropharynx, followed by the tonsils and tongue. Adequate knowledge about the characteristics of this virus and their association with the oral cavity is necessary, and could help oral health professionals to distinguish between different infections.

## 1. Introduction

Monkeypox (MP) is a rare disease caused by infection with a virus that is part of the same viral family as variola [1]. Monkeypox is zoonotic in origin and can spread from human to human [2]. It is part of the genus orthopoxvirus, which belongs to the poxviridae family. The first two outbreaks were accidental and occurred in monkeys kept for research in 1958, which was the year it was discovered [1]. Later on, its first manifestation in a human was in the Republic of Congo in 1970 [2]. The largest outbreak was between 1996 and 1997 in the same region [3]. Outside Africa, the first outbreak of monkeypox (MP) was in 2003 in the United States due to contact with infected prairie dogs [2]. Recently, it spread again worldwide in 2022, but almost exclusively among homosexual individuals. Generally, it is transmitted from human to human through direct contact with respiratory droplets, ulcerated lesions, and contaminated matter such as clothing, linens, bed sheets, and electronic devices [4,5]. Airborne and sexual transmissions have been a topic of controversy; however, there are numerous sources of evidence for its spread via sexual intercourse between men [6,7]. MP is most common in bisexual and gay men aged between 20 and 50 [6]. It begins with a non-contagious, asymptomatic, 7–14- or 5–21-day incubation period [8]. The contagious prodromal period later manifests with myalgia, lymphadenopathy, fever, headache, chills, and asthenia [8,9]. The initial lesions occur in the oropharynx preceding dermal rashes, mainly in the face, palms, and soles [10,11,12]. In the most recent outbreak, lesions first manifest in the anogenital and oral sites [13,14]. They typically evolve through multiple stages; macule, papule, vesicle, pustule, and contagious (as a crust) [15]. Complications such as encephalitis, pneumonia, and ocular lesions could arise [16]. Although fatalities have been reported in endemic cases, non-endemic ones have no reports of death [17]. Since the oropharynx is often the first site of these lesions, it is crucial to investigate such findings to provide immediate medical and dental attention. Oral health professionals need current knowledge of the characteristics and clinical manifestations of the numerous viruses that can inhabit the oral cavity and elsewhere in the upper aerodigestive tract. Dental practices could play an essential role in screening for endemic, and especially viral, infections, and in ensuring prompt referral for treatment, thus helping to mitigate further transmission. The current study aims to summarize the current evidence on the common oral and perioral manifestations of monkeypox, and to identify the most common locations and oral clinical presentations related to monkeypox.

## 2. Materials and Methods

The protocol for this study followed the evidence synthesis for case reports reported by Wiyeh et al. [18]. The study entailed an enquiry as to whether any preliminary research had been carried out with regard to the topic; the establishment of study selection criteria based on our search strategy, which included inclusion and exclusion criteria; charting the data; and collating and presenting the results. Studies were searched through the PubMed, Research Gate, and Wiley Online Library databases, and the Google search engine. The search used the keywords monkeypox; Mpox; poxvirus; zoonotic orthopox DNA virus; sore throat; ulcers; and vesicles. Of the 56 publications, 30 were selected, including 27 case-reports, two case series types, and one cross-sectional study. The studies were selected based on the inclusion of frequencies of specific locations and manifestations of oral lesions. The frequencies of descriptions of oral presentation and location in patients were calculated over the sample size and estimated as percentages. The descriptive statistics of these observations are demonstrated using bar charts. The exclusion of the rest of the 26 studies was due to the absence of data related to the frequency of oral/perioral manifestations and locations; the inability to access the studies; and large sample sizes in systematic reviews, observational studies, case series, and descriptive, retrospective, and prospective studies, which conflicted with the study.

## 3. Results

The total number of patients included in these studies was 54. Twenty-five of them were in the USA [19,20,21,22,23,24,25,26,27,28], three in Germany [10,29], six in Italy [30,31,32,33], one in the Republic of Congo [34], one in the Republic of Korea [35], two in India [36], three in the UK [37,38], one in Romania [39], one in the Czech Republic [40], two in France [41,42], six in Brazil [43,44,45], one in Portugal [46], and two in Columbia [47]. There were 43 (79.62%) males and 11 (20.37%) females. The age range was 3–69 years.

Of the 54 patients, 47 had oral/perioral manifestations. Twenty-three of them were in the USA [19,20,21,22,23,24,25,26,27,28], two in Germany [10,29], six in Italy [30,31,32,33], one in the Republic of Congo [34], one in the Republic of Korea [35], one in India [36], two in the UK [37,38], one in Romania [39], one in the Czech Republic [40], two in France [41,42], five in Brazil [43,44,45], one in Portugal [46], and one in Columbia [47].

**Figure 1:**
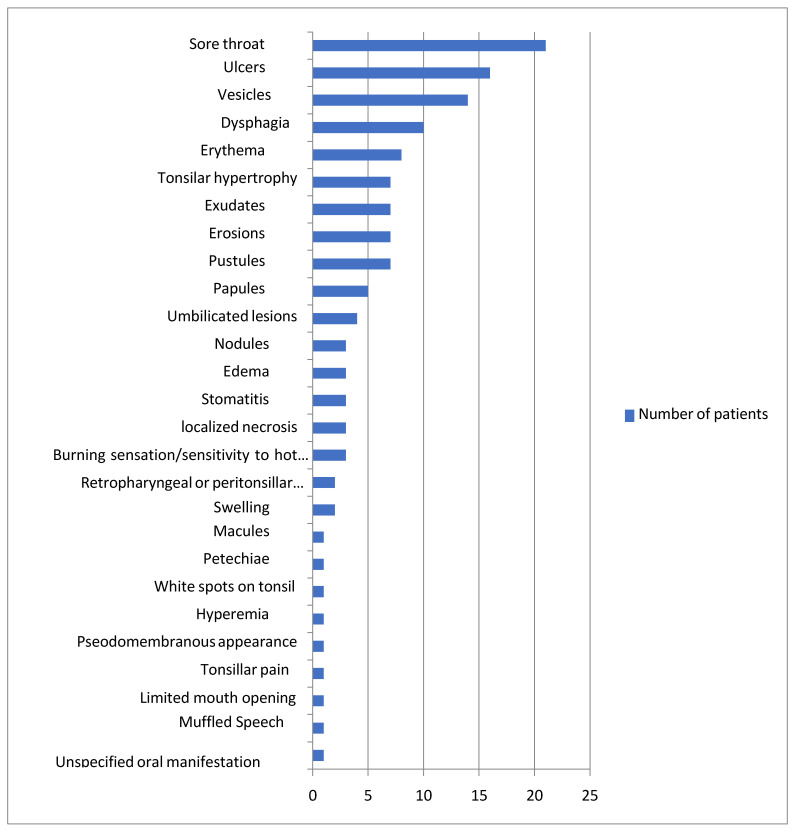
Prevalence of oral manifestations in MP patients in the selected studies.

Oral/perioral signs as one of the initial manifestations were reported in 23 out of 47 patients. Out of the 47 patients with oral/perioral involvement, the most frequent related sign/symptom was sore throat (n = 21), followed by ulcers (n = 16), vesicles (n = 14), dysphagia and odynophagia (n = 10), and erythema (n = 8). Then, tonsillar hypertrophy, exudates, erosions, and pustules were each reported in seven patients, papules in five patients, and umbilicated lesions in four patients. Nodules, edema, stomatitis, localized necrosis, and a burning sensation or sensitivity to hot food each manifested in three patients, and retropharyngeal or peritonsillar abscess and swelling were each reported in two patients. Macules, petechiae, white spots on the tonsils, hyperemia, a pseudomembranous appearance, tonsillar pain, limited mouth opening, and muffled speech due to swelling of the base of the tongue had the lowest occurrence (n = 1). An unspecified oral manifestation occurred in one patient [45] (Figure 1) (Table 1).

**Figure 2:**
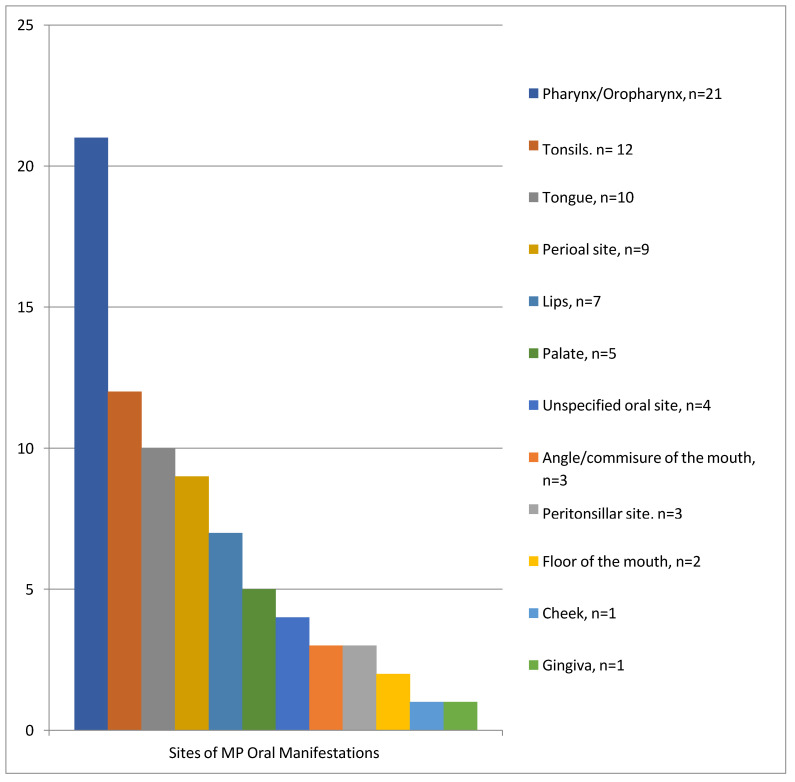
Prevalence of the sites of MP oral/perioral manifestations in patients of the selected studies.

The summary of the findings regarding the manifestation of oral/perioral sites demonstrates that the signs/symptoms were the highest in the pharynx/oropharynx (n = 21), followed by the tonsils (n = 12), tongue (n = 10), perioral site (n = 9), lips (n = 7), and palate (n = 5). The angles/commissures of the mouth and the peritonsillar site were affected in three patients, and the floor of the mouth in two patients. Finally, the cheek and gingiva had the lowest occurrence (n = 1). Lesions on the oral mucosa mentioned without specification of the site were represented in four patients [23,33,37,45] (Figure 2) (Table 1).

## 4. Discussion

### 4.1. Oral Manifestations

Since MP lesions usually begin orally, dentists are likely the first professionals to encounter them. The current paper aims to comprehensively characterize the most common presentation of MP in the mouth, which dental professionals must be aware of to diagnose and manage it accurately. This is the first attempt to evaluate the most common oral/perioral sites and the manifestation of MP disease. 

Our review found that the most common reported oral symptom of MP is a sore throat, while the most common oral or circumoral sign is an ulcer. In this study, the least common signs/symptoms are macules, petechiae, white spots on the tonsil, hyperemia, a pseudomembranous appearance, tonsillar pain, limited mouth opening, and muffled speech. Likewise, a recent systematic review and meta-analysis conducted in 2023, which investigated data from 4042 MP patients included in 19 collected studies, found that the most common oral manifestation was sore throat (39.96%), while mouth sores had a 24.9% prevalence, tonsillitis had 18.24%, and mouth rash had 17.99% [48]. While the previous study demonstrated that tonsillitis was one of the most common manifestations, the current paper oppositely reports that vesicles (29.78%), dysphagia and odynophagia (21.27%), and erythema (17.02%) demonstrated higher occurrence than tonsillar hypertrophy, a sign of tonsillitis (14.89%).

Patel et al. (2022) also found that amongst the 197 individuals infected with the MP virus in a case series study in the UK, sore throat had the highest oral occurrence (16.8%), while pustules, edema, abscess, or tonsillar erythema had a 4.6% prevalence [49]. Another publication in the UK based on 54 MP virus-infected individuals illustrated a prevalence of 20% of sore throat [50]. Catala et al. (2022), in Spain, in a prospective cross-sectional study on 185 MP patients, found that the most common oral manifestation was sore throat (18%), while oral ulcers had a 5% prevalence [51]. In another prospective publication in Spain in 2022 with a sample size of 181, sore throat was the highest oral finding (36%), followed by oral ulcers (25%) and tonsillitis (10%), which were accompanied by white ulcerated lesions in all of the cases [52]. Contrary to the present findings, a 2019 published cross-sectional study conducted in Nigeria found that oral sores were higher in prevalence (11 out of 21 MP patients) than a sore throat (9 out of 21 MP patients) [53]. Details from the selected studies reported in the current study suggest that the most frequent oral site where symptoms occur is the pharynx or oropharynx, primarily as a sore throat. The most common sites where signs occur are the tonsils, followed by the tongue. While no publication yet outlines the most common oral/perioral site where MP lesions occur, Patel et al. mentioned that 27 out of 197 MP patients in their study manifested oropharyngeal lesions without specification of the sites [49]. Thornhill et al., 2022, reported 66 patients who manifested oral involvement out of 528 MP patients. Of the 66 patients, 51 were found to have oropharyngeal lesions, but again, the site-wise emphasis on oral manifestations was not elaborated upon [54]. In a UK retrospective study of 54 MP-infected patients, oropharyngeal lesions were found in 4 patients (7%), two of them in the tongue. The locations of the other two lesions in the mouth were not mentioned [50]. In almost half of the cases included in the study, oral lesions were one of the initial signs of MP, while most patients had prodromal signs and symptoms such as sore throat, fever, malaise, and lymphadenopathy. Accordingly, it is worth considering MP as a differential diagnosis, especially if the affected patient is homosexual. Furthermore, the available studies have reported oro-facial presentations but have not characteristically focused on where these presentations are most prevalent. In our study, we tried to collate these findings and report the most prevalent areas in the oral region where the symptoms have been present, thereby providing room for observation for dentists and the healthcare professionals.

The vast range of viral infections that impact oral tissues, and the lower incidence of oral viral infections in ordinary dental practice, constitute a significant challenge in identifying oral viral infections rather than complicated clinical presentations. The early diagnosis of oral viral infections will lower clinical care costs, comorbidity, and morbidity. Oral lesions have the benefit of being easier to see in an examination. Blisters, ulcers, color fluctuation, surface/textural alterations, and other symptoms are simple to distinguish from those of other illnesses.

### 4.2. Differential Diagnosis

The initial clinical manifestations of MP can resemble the signs and symptoms of other diseases, such as the seasonal flu or the common cold; thus, being familiar with differential diagnosis is critical to rule out similar infections. Smallpox can be similar to MP in its clinical presentation; however, after the introduction of smallpox vaccines, it is no longer a threat to the general population [55]. MP can be often be misdiagnosed due to the similarities in its presentation to other viral and bacterial infections, especially in the oral cavity. The most common implication among these infections is a sore throat, a symptom of pharyngitis, and an inflamed oropharynx, particularly in the posterior aspect [56]. Monkeypox could be mistakenly diagnosed as viral or bacterial infections that present sore throat with or without oral lesions. Viral pharyngitis tends to occur seasonally, manifesting as coryza, fatigue, malaise, conjunctivitis, hoarseness, low-grade fever, mouth breathing, nausea, diarrhea, and abdominal pain. The associated oral lesions usually occur anteriorly as stomatitis [57]. Bacterial infections usually occur in the winter and early spring. The associated sore throat can feature cough, conjunctivitis, and high-grade fever, and usually, rhinorrhea is absent [58]. Suppurative and non-suppurative sequelae can follow bacterial pharyngitis. There is no evidence suggesting a longer duration of bacterial infections than viral ones or their higher severity in pharyngitis [57]. Generally, viral infections cause 70% of pharyngitis cases, while bacterial pharyngitis is present in 20–30% of cases [59]. The most notable viral infections include herpes simplex virus, varicella zoster virus, Epstein–Bar virus, and Coxsackie virus infections. Bacterial infections that could arise from sexual contact and present oral manifestations include syphilis and gonorrhea, while tuberculosis spreads through aerosol droplets. Scarlet fever, caused by Group A beta-hemolytic streptococcus, also manifests as oral lesions, but the organism is transmitted via direct contact [60]. Coinfections with other sexually transmitted diseases are common amongst MP patients, comprising 29% to 31.5% of cases. Gonorrhea and chlamydia are the most common types of accompanying infection with the MPXV, while herpes simplex virus accounts for 1–7% of cases [49,54]. The presence of coinfection may render the diagnosis of MP more challenging. Thus, proper laboratory tests and appropriate measures should be applied.

#### 4.2.1. Herpes Simplex Virus

Two types of herpes simplex virus may be similar in oral presentation to MP, and HSV-1 and -2. HSV-1 causes primary and secondary vesicular lesions, which, although they may present in the genital mucosa, affect mainly the oro-labial mucosa. HSV-2 is contrary to HSV-1, primarily affecting the genital area. HSV-1 and -2 are spread either via sexual intercourse or via direct contact [61]. MPXV is transmitted through direct contact, but although it is common among MSMs, it is not an STD. 

HSV-1 is commonly asymptomatic, but when there are symptoms, they usually manifest as fever blisters. In children, following fever and lymphadenopathy, the oral manifestations of HSV-1 infection are gingivostomatitis and vesiculo-ulcerative eruption on the perioral skin, vermillion border, and intra-oral mucosal surface. The vesicles, after rupture, are painful as they are left to heal without scarring [62]. The herpes simplex virus migrates in later stages to the trigeminal ganglion, accounting for 15% to 30% of cases, and is affected by the reactivation of secondary herpes simplex lesions in early adulthood due to stress or infection. Notable ulcerated vesicles on the lip, known as herpetic labialis, usually preceded by a burning sensation in the area, are present at this stage. In adults not previously infected with HSV-1, the oral presentation includes viral pharyngitis with ulcers on the posterior pharynx and tonsils, and cervical lymphadenopathy [63].

While primary herpetic gingivostomatitis presents similar lesions to MP, they are limited to children, unlike MP, which is more likely in adults. Secondary herpetic gingivostomatitis is always present in adulthood, so the diagnosis here is more challenging. Lesions at this stage are confined to keratinized, attached mucosa, whereas oral lesions in MP are widespread. In addition, properly establishing the sexual history of MSM (men who have sex with other men) can rule out MP in secondary herpetic infections.

In HSV-2 infection, lesions most often occur at the mucocutaneous junction on the lip or perioral skin. They comprise a small cluster of vesicles that enlarge, combine, become ulcerated, and develop a crust before healing within ten days. The lesions in the mouth represent a painful group of vesicles on an erythematous base with a scalloped border [63]. They progress to pustules, erosions, and ulcerations. Those lesions in HSV are commonly present at the same anatomical site in different stages, which is dissimilar to the progression of rash in monkeypox virus, which starts with macules and develops into papules, vesicles, and pustules, followed by crusting. Both infections are viral; thus, the treatment of choice is antiviral medication such as acyclovir or tecovirimat. The use of acyclovir for HSV is the treatment of choice and will reduce the severity of the symptoms, but does not cure the infection or prevent its recurrence [64]. The use of tecovirimat for MP has been shown to be effective with viral clearance, showing negative oropharyngeal, rectal, and skin swab results [65]. 

#### 4.2.2. Varicella Zoster Virus

Varicella, also known as chickenpox, is an acute infectious disease. After the primary infection of VZV, the virus stays in the body in the dorsal spinal ganglia or trigeminal ganglion as a latent infection that, through reactivation, causes shingles. During their early stages, MPXV and VZV have similar anatomical appearances in the macule and papule. The critical factor in establishing the difference involves immaculate attention to the onset and progression of the diseases to establish a diagnosis. Fever in both diseases has a different pattern. In VZV, low-grade fever is present at the onset of the rash, whereas MP patients experience high fever 1–4 days before the onset of the rash. In VZV infections, intraoral vesicles are typically located on the buccal mucosa, palate, tongue, gingival mucosa, and oropharynx. The vesicles appear on only one side of the head and neck region and do not cross the midline of the affected area [63]. The vesicles ulcerate and form pustules within 3–4 days. Then, a crust forms and heals within 7–10 days. The most crucial clinical indicator used to differentiate between secondary VZV infections and MPXV infections is the location of the vesicles, as they are present unilaterally in the former and have no specific pattern in the latter. Another indicator is the absence of lymphadenopathy in VZV infections, while it occurs in monkeypox [66,67]. 

#### 4.2.3. Epstein-Barr Virus

Epstein-Barr virus is related to compromised immunity and is associated with AIDS. Oral hairy leukoplakia is a distinctive characteristic of EBV, and is clinically present as raised white areas that commonly occur only on the lateral border of the tongue. They are typically painless, and thus, can be unnoticed [63,68]. However, although HIV patients are also susceptible to MP infection, MP lesions on the tongue are acute and painful and usually present on the tongue’s ventral surface, its tip, and its dorsum. This infection necessitates a biopsy swab to confirm it. A positive EBV result indicates a positive diagnosis. No drug has been found to be effective for treating oral hairy leukoplakia, and although many agents have been tested, antiviral therapy was rendered ineffective [69,70]. On the other hand, tecovirimat is effective in treating MP [65].

#### 4.2.4. Coxsackie Virus

Different strains of this virus cause different diseases. Coxsackie A16 and enterovirus A71 are responsible for causing hand, foot, and mouth disease [71,72,73,74]. This disease affects children below ten years of age. Fever, pharyngitis, and sore throat are common symptoms. After the onset of fever, within 1 to 2 days, lesions appear in the mouth and throat. Other lesions may appear on the hands, feet, mouth, buccal mucosa, knees, elbows, and buttocks. Oral lesions appear as vesicles that rapidly ulcerate, forming multiple small superficial ulcers with an erythematous halo [72,75]. These ulcers are found on the tongue, gingiva, lips, buccal mucosa, and palate. The duration of rash in monkeypox is about two times that in hand, foot, and mouth disease (HFMD), accounting for 14–28 days in the former and 7–10 days in the latter [76,77]. A careful history and laboratory assessment can help distinguish between MP and HFMD. Lymphadenopathy is common in MP, unlike HFMD [78]. The progression of the rash can be an indicator, as well. MP lesions progress as macules, papules, vesicles, pustules, and scabs. HFMD progresses as macules, and then, vesicles, which may progress to scabs. 

Another disease caused by the Coxsackie virus is herpangina. It is caused by 22 enterovirus serotypes and is mainly associated with the Coxsackie B virus serotype [79,80]. Painful enanthem is usually present on the soft palate, posterior pharynx, and tonsils. Herpangina is characterized by discrete erythematous macules that turn into vesicles, and eventually, a center ulcer, typically found on the soft palate, the tonsillar area, the buccal wall, and the posterior third of the tongue [80]. They persist for about seven days. Similarly to monkeypox, herpangina symptoms include high fever. HFMD and herpangina are self-limiting and only require supportive care. 

#### 4.2.5. Syphilis

Treponema pallidum is the etiological agent responsible for the rise of syphilis. This sexually transmitted disease has four overlapping stages: primary, secondary, latent, and tertiary [81]. Since both syphilis and MP are diagnosed through sexual history, it is challenging to conclude a final diagnosis. It is even more difficult to confirm the current presence of either infection, while a medical history establishes previous lesions in a syphilis patient. Oral manifestations are the first signs of primary, secondary, and tertiary syphilis. In primary syphilis, oral lesions known as a chancre commonly appear and are painless, solitary, and round, with indurate ulceration and firm margins. Hyperplasic foliate papillae and asymmetry of the uvula are accompanying signs [82,83].

Lesions at this stage, usually similar to MP lesions, begin as macules and progress into papules that eventually ulcerate. Petechial hemorrhage in the palate and lymphadenopathy are also possible signs [84,85,86,87]. Secondary syphilis oral lesions present as macular and papular eruptions or mucous patches. They are typically slightly raised erosions or shallow ulcers with erythematous borders, covered with an overlying gray pseudo-membrane [88]. The oral lesions are painful at this stage. Fever, sore throat, and lymphadenopathy are common. In tertiary syphilis, oral lesions are more severe and present on the hard palate. They can proliferate into bone, reaching the nasal septum. Those on the tongue, called leukoplakia, make it fissured and atrophic. MP symptoms are similar to secondary syphilis presentations, including rash, fever, headache, pharyngitis, and lymphadenopathy. In secondary syphilis, rash progression can present as papular, annular, or pustular and have a grey pseudo membrane. However, the rash progression in MP follows a different pattern: progression from macules to papules, vesicles, and pustules, followed by scabbing and desquamating. A serological investigation confirms the diagnosis [88].

#### 4.2.6. Gonorrhea

Neisseria gonorrhoeae is a sexually transmitted Gram-negative coccus that infects only humans, and is the causative pathogen of Gonorrhea disease. Gonorrhea that affects women’s mucous membranes causes cervicitis, and in men, it causes urethritis [89]. In a case report study of 245 patients, in which gonorrhea was reported in 64 patients, the prevalence of pharyngeal gonorrhea in MSM was 2–11%, in heterosexuals, 3–7%, and in women, 2–10%. This study also concluded that pharyngeal gonorrhea occurs mainly in those who perform oral sex [90]. Persistent sore throat is the most prominent symptom of pharyngeal gonorrhea. In MP, oral lesions are prominent and not limited to the oropharynx, which is not the case for gonorrhea. 

#### 4.2.7. Scarlet Fever

Patients who develop streptococcal skin or wound infections could develop scarlet fever. The causative factor is a Gram-positive A beta-hemolytic streptococci group called Streptococcus pyogenes. This disease can be transmitted via nasal secretions or infected saliva. It is most common among children aged 5 to 15 [91,92]. Since non-endemic MP is now prevalent, infected patients are usually middle-aged MSM [6]. The strawberry tongue is the most common oral manifestation of scarlet fever, characterized by hyperplastic fungiform papillae with a white coating. When the white layer is scraped, the tongue appears raw and red with a bumpy appearance [93]. Oral scarlet thrush commonly does not accompany ulcerated lesions. Oral thrush in MP possibly accompanies a rash [22]. The throat in scarlet fever, called strep throat, also appears erythematous with yellowish patches, resulting in pain when swallowing. A throat culture is the most appropriate method of detecting this bacterial infection [56]. However, in MP, a PCR swab is performed [94]. Scarlet fever requires antibiotic treatment; otherwise, the infection might reoccur or progress into rheumatic fever after about 1–5 weeks, and post-streptococcal glomerulonephritis is a possible sequela [58,95]. Mild symptoms of MP might resolve with or without supportive therapy [20,28,31,45].

#### 4.2.8. Tuberculosis

Mycobacterium Tuberculosis is the causative agent of tuberculosis [60]. Oral manifestations of tuberculosis rarely occur, yet it has been considered to account for 0.1–5% of all tuberculosis infections. Lesions in the oral cavity can be of two types according to their occurrence: primary or secondary. Primary TB lesions are uncommon and appear as a single painless ulcer with regional lymph node enlargement. Secondary lesions are more common, and are typically associated with pulmonary disease. Oral lesions in secondary TB describe single, indurated, irregular, painful ulcers covered with inflammatory exudates [60,96]. On the contrary, oral lesions in MP do not appear as single lesions; they are commonly multiple lesions. Tuberculosis lesions may appear at any location in the oral cavity, but mainly on the tongue. The tonsils, uvula, and salivary glands are also commonly involved [97]. Typically, a TB primary lesion appears along the lateral border of the tongue that rests against areas of continuous irritation, such as sharp, rough, or broken teeth. The areas with constant trauma and irritation can render the tongue more susceptible to the localization of Mycobacterium [98]. While MP is detected through a PCR swab, tuberculosis is detected through a blood or skin swab [94,99].

#### 4.2.9. Management of Monkeypox

In terms of the management of MP, there is yet to be an approved drug; multiple treatment and management options have been tried and tested, including tecovirimat, an antiviral that was effective in all MP patients in several studies [19,21,22,29]. In one case series included in this review, patients’ lesions resolved in 2–4 days after taking a 600 mg dose of tecovirimat twice daily [21]. Signs of the drug’s effectiveness included a reduction in size or expansion of the lesions, or their crusting [21,22]. Although one of the patients developed additional pustules on the gingiva during the second day post-drug intake and on the extremities during the third day, gradual healing of the odynophagia and tonsillar edema was observed. The side effects of tecovirimat were mild headache after the first dose in one case, and loose bowel movements in another [21]. Another antiviral drug deemed effective is acyclovir, administered intravenously, which ceased the development of new lesions and led to the crusting of vesicles [23]. The concomitant intake of amoxicillin, chlorhexidine mouthwash, and systemic and local analgesics such as acetaminophen and xylocaine were found to be effective [42]. In other cases, supportive therapy was rendered sufficient to heal the lesions [28,31]. Antibiotics and antifungals should be prescribed cautiously after ruling out unfavorable diagnoses. Otherwise, a secondary infection could exacerbate the condition, leading to progressive oral lesions, cellulitis, sepsis, other complications, or even death. [20,26,34,40]. If severe oral lesions have limited oral intake, it is prudent to maintain hydration by providing fluid [34,39].

According to the CDC, lesions involving the pharynx and cause dysphagia necessitate treatment, primarily when parenteral feeding is implemented or when seen in high-risk patients such as those infected with HIV [100]. The treatment of oral monkeypox lesions is mainly supportive. Pain management, oral antisepsis, fluid maintenance, and nutritional support play a role in this approach. Pain can be managed with an analgesic mouthwash (magic mouthwash), which contains an antihistamine or an anesthetic, salt water mouth rinse four times a day, and a local anesthetic (viscous lidocaine). Oral antiseptics, such as chlorhexidine mouthwashes, and adherence to oral hygiene measures are recommended to prevent bacterial superinfection or the risk of transmitting the disease. Antivirals are considered in severe cases [101,102]. Although cidofovir was shown to be successful in treating severe cases, its effect against oral lesions is poorly documented [100].

Though the CDC have reported guidelines related to the management of MP, few studies have adequately reported its oral presentations and management at the time of writing this study. The limited number of reports on oral manifestations in the form of both case reports and case series prevents the recommendation of standardized treatment guidelines, which highlights the necessity for trials of superior quality and magnitude. The possibility of viral mutations, furthermore, can risk the presence of these presentations in areas where they are otherwise not reported. Indeed, a holistic approach towards the management of oral and systemic lesions is likely to be more meaningful.

## 5. Conclusions

Oral manifestations are valuable indications for raising suspicion of MP. Based on the current findings, it has been found that oral lesions predominately occur in the pharynx/oropharynx, tonsils, and tongue. Dentists must consider MP in their differential diagnoses of patients with sore throat, oral ulcers, vesicles, dysphagia, and odynophagia, especially when skin lesions, fever, malaise, and lymphadenopathy accompany these symptoms. Dentists need to be prepared and diligent in their approach towards patients exhibiting such symptoms. Timely interventions in the form of investigations and referrals for secondary opinions should be the norm before treating such cases. This mitigates the risk of spreading this zoonotic disease.

## Figures and Tables

**Table 1:** Data collected on studies describing oral/perioral manifestations in MP patients.

Author and Year of Publication	Study Design	Country	Number of Patients	Gender	Age	Number of Patients with Oral/Perioral Manifestations	Oral/Perioral Manifestation	Oral/Perioral Manifestation Site	Treatment	Oral or Perioral Manifestation as One of the Initial Signs/Symptoms of the Disease
Anderson et al., 2003 [24]	Case report	USA	1	Female (1)	School age	1	Red macules, vesicles, retropharyngeal phlegmon, dysphagia, and sore throat	Macules in the mouth and pharynx, vesicles on the tongue and peritonsillar area, abscess in the retropharyngeal area, and sore throat (pharynx)	IV ampicillin/sulbactam	No
Reed et al., 2004 [25]	Case series	USA	11	Male (5), Female (6)	3–43 years old	10	Sore throat: 55% (n = 6), Pharyngitis: 27% (n = 3), tonsillar hypertrophy: 18% (n = 2), tonsillar erosions: 18% (n = 2), and other lesions in the buccal mucosa (frequency was not mentioned)	Pharynx (n = 6), tonsils (n = 4), and lesions found on the buccal mucosa (frequency was not mentioned)	IV acyclovir, valacyclovir	No
Sejvar et al., 2004 [20]	Case series	USA	3	Male (1), Female (2)	33 years old (male)	3	Sore throat	Pharynx	No medical care was provided	No
					30 years old (female)		Papule and sore throat	Papule in the cheek, and sore throat (pharynx)	Bolus of Prednisone, resulting in subsequent improvement in dyspnea	Yes
					6 years old (female)		Enlarged tonsils; pharyngeal erythema and edema; profuse, thick exudates; and sore throat	Tonsils and pharynx	Antimicrobial treatment (cefdinir), antipyretics, lorazepam, IV ceftriaxone, IV acyclovir, IV phenobarbital, and IV midazolam	No
Vaughan et al., 2018 [37]	Case report	UK	2	Male (2)	Middle-aged	1	Vesicles	Vesicles on the mucosal surface of the mouth	NM	No
					Middle-aged		No oral/perioral manifestation	No oral/perioral manifestation	NM	No
Eltvedt et al., 2020 [34]	Case report	Republic of Congo	1	Male (1)	4 years old	1	Stomatitis and vesiculopapular rash	Vesiculopapular rash on the lips	IV amoxicillin–clavulanic acid, retinol tablets, antibiotic eye drops, and paracetamol. Then, changed to IV (ceftriaxone) and morphine.	No
Costello et al., 2022 [23]	Case report	USA	1	Male (1)	28 years old	1	Erosions and a pustule	Erosions on oral mucosa and a pustule in the lower mucosal lip	IV acyclovir	No
Noe et al., 2022 [10]	Case report	Germany	2	Male (2)	26 years old	1	White spots and dysphagia	White spots on tonsils	Topical zinc oxide suspension	Yes
					32 years old		No oral/perioral manifestation	No oral/perioral manifestation	Topical zinc oxide suspension	No
Jang et al., 2022 [35]	Case report	Republic of Korea	1	Male (1)	34 years old	1	Erosions covered with crust and sore throat	Erosions in the perioral area and sore throat (pharynx)	NM	Yes
de Sousa et al., 2022 [46]	Case report	Portugal	1	Male (1)	24 years old	1	Umbilicated papule and ulcer	An umbilicated papule on the upper lip and an ulcer on the dorsal surface of the tongue	Symptomatic care with paracetamol and tramadol, and topical antibiotic (fusidic acid)	No
Ajmera et al.,2022 [22]	Case report	USA	1	Male (1)	26 years old	1	Rash as tender umbilicated pox-like lesions/papules, swelling, sore throat, pain on swallowing, burning sensation, and oral thrush	Rash (umbilicated lesions) on the tongue and perioral rash (umbilicated pox-like lesions), swelling of the tongue, and sore throat (pharynx)	Antibiotic therapy (IV vancomycin, IV piperacillin), IV dexamethasone, IV acyclovir and IV fluconazole, magic mouthwash, Valtrex, IM penicillin, tecovirimat, and supportive care	Yes
Yadav et al., 2022 [36]	Case report	India	2	Male (2)	35 years old	1	Umbilicated vesicular rashes, edema, and sore throat	Vesicular rashes found in the oral cavity (tip of the tongue) and lips, edema of the upper lip, and sore throat (pharynx)	Oral acyclovir	Yes
					31 years old		No oral/perioral manifestation	No oral/perioral manifestation	NM	No
Schlabe et al., 2022 [29]	Case report	Germany	1	Male (1)	51 years old	1	Ulcer, vesicles, swelling, and muffled speech	Vesicles turned ulcers on the left mouth corner/commissure, vesicles on the palate, and swelling of the base of the tongue leading to muffled speech	Antiviral medication (tecovirimat)	Yes
Ortiz-Martínez et al., 2022 [26]	Case report	USA	1	Male (1)	36 years old	1	Sore throat, bilateral enlarged tonsils, and oropharyngeal erythema	Sore throat and erythema (oropharynx), and tonsils	IM penicillin G, doxycycline, ceftriaxone, and amoxicillin–clavulanate	Yes
Davido et al., 2022 [41]	Case report	France	1	Male (1)	48 years old	1	Limited mouth opening, abscess, and swallowing disorder	Peritonsillar abscess on clinical examination, but CT scan revealed peritonsillar abscess swelling in the piriform sinus	Antimicrobial therapy (IV amoxicillin/clavulanate, IV ceftriaxone, and oral metronidazole) and drainage	Yes
Matias et al., 2022 [21]	Case report	USA	3	Male (3)	20s	2	Pruritic vesiculopustular lesions	Oropharynx	Antiviral therapy (tecovirimat)	No
					20s		Tonsillar enlargement and pain, edema, pustular lesions, and odynophagia	Enlargement of the left palatine tonsil, and pustular lesions on the gingiva	Antiviral therapy (tecovirimat)	Yes
					40s		No oral/perioral manifestation	No oral/perioral manifestation	Antiviral therapy (tecovirimat)	No
Lima et al., 2022 [43]	Case report	Brazil	1	Male (1)	41 years old	1	The lesions followed a vesicle–pustule–ulcerated lesion pattern with a well defined border and a central crust surrounded by erythema, along with another ulcer	The lesions that followed the common pattern were seen above the upper lip, while the ulcerated lesion was in the oropharynx	Antiviral therapy (valaciclovir), antibiotics (doxycycline, azithromycin, ceftriaxone, and amoxicillin–clavulanate), and antipyretics (dipyrone)	Yes
Lopes et al., 2022 [44]	Case report	Brazil	2	Male (2)	28 years old	2	Ulcero-crusted lesion with vesiculopustular borders and necrotic, exudative background, and localized erythema	Rima oris/angle of the mouth	Paracetamol and cleaning of the lesions with antiseptics	Yes
					28 years old		Lesion with vesiculopustular borders, central necrotic tissue, and an erythematous base	Rima oris/angle of the mouth	Paracetamol and cleaning of the lesions with antiseptics	Yes
Eisenstadt et al., 2022 [27]	Case report	USA	1	Male (1)	20s	1	Sore throat and honey-colored, superficial crusted ulcer	Sore throat (pharynx) and an ulcer on the perioral skin	2% mupirocin ointment for presumed impetigo and 5% imiquimod cream for presumed condyloma	Yes
Oprea et al., 2022 [39]	Case report	Romania	1	Male (1)	26 years old	1	Hyperemia of the pharynx with pseudo-membranous appearance, petechiae, thrush, and dysphagia	Hyperemia in the pharynx and petechiae on the palate	Symptomatic treatment, fluids, and topical treatment	No
Bížová et al., 2022 [40]	Case report	Czech Republic	1	Male (1)	34 years old	1	Ulcer	Ulcer on the left tonsil	Cephalosporins	Yes
Peters et al., 2022 [19]	Case report	USA	2	Male (2)	38 years old	2	Sensitivity to hot food, ulcer, and vasculo-ulcerative lesions	Sensitivity to hot food in the tongue, ulcer at the tip and midline of the tongue, and vasculo-ulcerative lesions on the anterior ventral tongue surface	Antiviral medication (tecovirimat)	Yes
					30 years old		Nodule, ulcer, and sore throat	Nodule on the tip of the tongue, ulcer on the anterior dorsal surface of the tongue, and sore throat (pharynx)	NM	Yes
Wong et al., 2022 [28]	Case report	USA	1	Male (1)	52 years old	1	Odynophagia, and tonsillar hypertrophy with exudates. Vesicles and sore throat (pharyngeal pain)	Bilateral tonsillar hypertrophy with exudates, vesicles on the pharynx, and sore throat	Supportive therapy	Yes
Benslama et al., 2022 [42]	Case report	France	1	Male (1)	34 years	1	Canker sores (ulcers) (target/cockade-shaped) surrounded by white halo, and difficulty swallowing	Ulcers at the tip of the tongue and floor of the mouth	Antimicrobial therapy (amoxicillin), local antiseptics (chlorhexidine), and general and local analgesics (paracetamol, xylocaine).	Yes
Lucer et al., 2022 [47]	Case report	Columbia	2	Male (2)	26 years old	1	Pruritic lesions in the mouth, ulcer, and pustules	Ulcer in the lower lip, pustules in the right soft palate	Supportive therapy, opiods for rectal pain, and tecovirimat	No
					37 years old		No oral/perioral manifestation	No oral/perioral manifestation	Opiods for rectal pain and tecovirimat	No
Pisano et al., 2022 [30]	Case report	Italy	2	Male (2)	45 years old	2	Dysphagia, sore throat, ulcerated nodule, erosive lesion with erythema, and enlarged tonsil with exudates deviating the uvula	Sore throat (pharynx), ulcerated nodule on the lateral border of the tongue, erythematic erosive lesion on the palate deviating the uvula, and enlarged tonsils	NM	Yes
					69 years old		Ulcer and sore throat	Ulcer on the floor of the mouth and sore throat (pharynx)	NM	Yes
Pisano et al., 2022 [32]	Case report	Italy	1	Male (1)	54 years old	1	Nodules covered with scaly crusts with burning sensation	Lesions present in the perioral region	Systemic amoxicillin–clavulanic acid, and 2% fusidic acid ointment	No
Martin-Filho et al., 2022 [45]	Cross-sectional study	Brazil	3	Male (1), Female (2)	28 years old (female)	2	Sore throat and oral lesions (unspecified type)	Sore throat (pharynx) and lesions in the oropharynx, oral mucosa, and lips	No medical care was needed	Yes
					24 years old (male)		Sore throat	Sore throat (pharynx)	No medical care was needed	No
					12 years old (female)		No oral/perioral manifestation	No oral/perioral manifestation	No medical care was needed	No
Ambrogio et al., 2022 [31]	Case report	Italy	2	Male (2)	39 years old	2	Exudative erythematous plaque covered with vesicles, ulcer with central erosive area	Exudative erythematous plaque covered with vesicles on the chin, ulcer with central erosive area on the lower lip	Supportive therapy	No
					NM		Exudative ulcer	Exudative ulcer on the chin	Supportive therapy	No
Crosato et al., 2023 [33]	Case report	Italy	1	Male (1)	46 years old	1	Vesicle, followed by erosion, expansion, ulceration, and necrosis with umbilication of the lesion, and there was a papulo-vesicular rash	Ulcerated vesicle on the chin and papulo-vesicular rash in the oral cavity	Biopsy of the chin lesion, isolation of the patient, and no treatment was performed	Yes
Amos et al., 2023 [38]	Case report	UK	1	Male (1)	40s	1	Sore throat, odynophagia, hoarse voice, erythema, thick white exudates, vesicle, and ulcers	Sore throat (pharynx), bilateral peritonsillar erythema, right tonsillar exudates, vesicle in the left posterior palate, white patches of exudates and ulcers on the right base of the tongue	Nebulized epinephrine, IV dexamethasone, IV broad-spectrum antibiotics, IV fluids, analgesia, heparin, and oral anticoagulant	Yes

## Data Availability

No new data were created.
